# Identifying Patients Who Meet Criteria for Genetic Testing of Hereditary Cancers Based on Structured and Unstructured Family Health History Data in the Electronic Health Record: Natural Language Processing Approach

**DOI:** 10.2196/37842

**Published:** 2022-08-11

**Authors:** Jianlin Shi, Keaton L Morgan, Richard L Bradshaw, Se-Hee Jung, Wendy Kohlmann, Kimberly A Kaphingst, Kensaku Kawamoto, Guilherme Del Fiol

**Affiliations:** 1 Veterans Affairs Informatics and Computing Infrastructure Department of Veterans Affairs Salt Lake City Health Care System Salt Lake City, UT United States; 2 Division of Epidemiology, Department of Internal Medicine School of Medicine University of Utah Salt Lake City, UT United States; 3 Department of Biomedical Informatics University of Utah Salt Lake City, UT United States; 4 Department of Emergency Medicine University of Utah Salt Lake City, UT United States; 5 College of Nursing University of Utah Salt Lake City, UT United States; 6 Department of Population Health Sciences University of Utah Salt Lake City, UT United States; 7 Huntsman Cancer Institute University of Utah Salt Lake City, UT United States; 8 Department of Communication University of Utah Salt Lake City, UT United States

**Keywords:** clinical natural language processing, family health history extraction, cohort identification, genetic testing of hereditary cancers

## Abstract

**Background:**

Family health history has been recognized as an essential factor for cancer risk assessment and is an integral part of many cancer screening guidelines, including genetic testing for personalized clinical management strategies. However, manually identifying eligible candidates for genetic testing is labor intensive.

**Objective:**

The aim of this study was to develop a natural language processing (NLP) pipeline and assess its contribution to identifying patients who meet genetic testing criteria for hereditary cancers based on family health history data in the electronic health record (EHR). We compared an algorithm that uses structured data alone with structured data augmented using NLP.

**Methods:**

Algorithms were developed based on the National Comprehensive Cancer Network (NCCN) guidelines for genetic testing for hereditary breast, ovarian, pancreatic, and colorectal cancers. The NLP-augmented algorithm uses both structured family health history data and the associated unstructured free-text comments. The algorithms were compared with a reference standard of 100 patients with a family health history in the EHR.

**Results:**

Regarding identifying the reference standard patients meeting the NCCN criteria, the NLP-augmented algorithm compared with the structured data algorithm yielded a significantly higher recall of 0.95 (95% CI 0.9-0.99) versus 0.29 (95% CI 0.19-0.40) and a precision of 0.99 (95% CI 0.96-1.00) versus 0.81 (95% CI 0.65-0.95). On the whole data set, the NLP-augmented algorithm extracted 33.6% more entities, resulting in 53.8% more patients meeting the NCCN criteria.

**Conclusions:**

Compared with the structured data algorithm, the NLP-augmented algorithm based on both structured and unstructured family health history data in the EHR increased the number of patients identified as meeting the NCCN criteria for genetic testing for hereditary breast or ovarian and colorectal cancers.

## Introduction

### Background

Cancer screening has been shown to effectively reduce mortality [1,2]. Unlike population-based screening recommendations that target a broad range of individuals, increasing evidence supports individualized cancer screening according to cancer risk [3-5]. Individuals at higher risk may benefit from earlier, more frequent, or more intensive screening. Effective interventions are needed to stratify patients by risk and to direct them to an appropriate level of screening. However, individualizing screening on a population scale requires patient-specific risk assessments for several types of cancer. This is quite challenging in today’s overwhelmed primary care environment, as the current screening process requires manual chart review to identify patient candidates for genetic testing, and primary care providers often do not have time or knowledge to discuss genetic testing with their patients. A promising solution is to automate the identification of high-risk patients using electronic health records (EHRs) coupled with clinical decision support (CDS) tools.

The National Comprehensive Cancer Network (NCCN) has published a set of evidence-based guidelines for genetic testing of hereditary cancers, including breast, ovarian, pancreatic, and colorectal cancers [6,7]. A summary of these 2 guidelines is listed in Textbox 1, where each table cell represents a criterion, and the criteria for the same cancer cohort are listed in the same column. When one or more criteria are met, the corresponding genetic testing is recommended. These cancer risk assessment guidelines are based mainly on the family health history (FHH) of cancer or cancer syndromes, which is recorded in EHR systems as part of routine patient care activities. Therefore, EHR is one of the most important sources of FHH that can be used to drive CDS tools to help identify candidates for genetic testing of hereditary cancers [8]. However, several challenges limit the systematic use of FHH in EHR for these purposes, including (1) scattered FHH documentation in both structured and unstructured formats across different EHR sections, such as the clinical note [9], problem list, and FHH sections; (2) conflicting documentation in different sections of the EHR; (3) incomplete documentation in structured FHH data; (4) negation and ambiguity of information in unstructured data [10-12].

**Excerpt of National Comprehensive Cancer Network (NCCN) criteria for unaffected individuals’ family history–based genetic testing of breast, ovarian, pancreatic, and colorectal cancers (referenced with permission).**

**Breast or ovarian cancer:**

1. First- or second-degree relative with breast cancer at age ≤45 years
2. First- or second-degree relative with ovarian cancer
3. First-degree relative with pancreatic cancer
4. Breast cancer in a male relative
5. Three or more first- or second-degree relatives with breast or prostate cancer on the same side of the family
6. Ashkenazi Jewish and any breast or prostate cancer in any relative at any age
7. BRCA1/2, CHEK2, ATM, PALB2, TP53, PTEN, or CDH1 genes, Cowden Syndrome, Li-Fraumeni Syndrome in any relative at any age

**Colorectal cancer:**

1. MLH1, MSH2, PMS2, MSH6, EPCAM, MYH, or MUTYH genes, Lynch syndrome, familial adenomatous polyposis (FAP), adenomatous polyposis coli (APC), serrated polyposis or polyposis discovered in the coded family history
2. First-degree relative with colon cancer at ≤50 years
3. First-degree relative with endometrial cancer at ≤50 years
4. Three or more first- or second-degree relatives with Lynch syndrome, HNPCC, colon cancer, endometrial, uterine, ovarian, stomach, gastric, small bowel, small intestine, kidney, ureteral, bladder, urethra, brain, pancreas, also all on the same side of the family

Genetic testing for breast, ovarian, or colorectal cancer is recommended if at least one of these criteria is met.

Current EHR systems often provide a dedicated FHH section, in which FHH assertions can be captured using a combination of structured (eg, coded disease, relationship, and age of onset) and unstructured data (ie, the comments field). FHH free-text comments are different from broader clinical notes in that the former are associated with a specific structured FHH assertion, only available in the FHH section, while clinical notes can capture a much wider range of information, including medical history, physical examination, and treatment plans. Health care providers typically use free-text FHH comment fields when desired information cannot be fully captured as structured data. For example, a patient’s sister who developed breast cancer in her 30s can be captured partially as structured data (ie, condition = *breast cancer* and family member = *sister*) supplemented by a comment captured in the unstructured data conveying the uncertain age of onset (ie, *onset in her 30s*). The FHH section is increasingly used as part of routine visit intake by medical assistants and by patients themselves through patient portals [13]. Therefore, the FHH section is a promising and underused source of FHH for EHR.

Previous studies have largely focused on extracting FHH from clinical notes [14,15]. This study is the first comprehensive attempt to supplement structured FHH data with information extracted from free-text comments. The natural language processing (NLP) extraction of information from free-text comments imposes a unique set of challenges that require specific approaches that have not been investigated. Specifically, candidate approaches must address the interplay between structured and unstructured data collected in the FHH section.

### Objectives

Our previously developed structured algorithm [8] for identifying patients who met the NCCN criteria for genetic testing using structured data demonstrated the potential use of this dedicated FHH section. Nonetheless, we noticed that the algorithm based on structured data failed to correctly identify certain cases because some information needed for eligibility determination was recorded as free-text comments. For example, an FHH entry included *CANCER* and *AUNT* as structured data, with the specific type of cancer and age of onset (*breast ca, dx in 30s*) provided as a free-text comment. This case would be considered eligible for genetic testing when using the information provided in the comments section. These errors resulting from the structured data algorithm added a manual review burden for genetic counseling staff because they needed to manually confirm patient eligibility before communicating with them.

Hence, this study aims to augment CDS algorithms that rely exclusively on structured FHH data with information extracted from free-text FHH comments fields using NLP, with a focus on identifying patients who meet the NCCN criteria for genetic testing for hereditary breast or ovarian and colorectal cancers. The corresponding NLP was designed to extract the FHH information when it was not available or accurately coded in structured data, including the cancer type (eg, pancreatic cancer), the age of onset (eg, in the early 30s), and the affected family member (eg, *paternal aunt*). The primary hypothesis is that using NLP to augment the previously developed algorithm (using structured data alone) [8] can improve the accuracy of identifying patients who meet the NCCN criteria for genetic testing based on the FHH of patients seen in primary care settings at a US academic medical center.

## Methods

### Study Design

**Figure 1:**
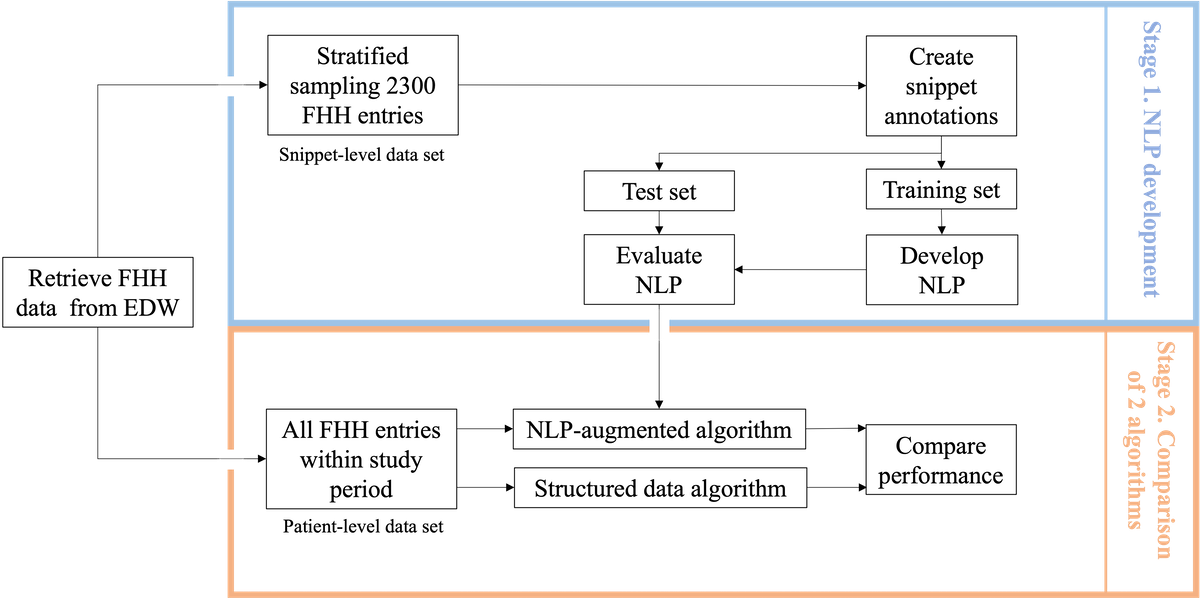
Study stages, including natural language processing (NLP) development (stage 1) and comparison between the NLP-augmented algorithm and an algorithm using only structured data (stage 2). EDW: enterprise data warehouse; FHH: family health history.

We retrospectively studied data from the EHR at the University of Utah Health. The study consisted of 2 stages (Figure 1). In the first stage, for NLP development, an NLP solution was developed to extract FHH information from both structured and unstructured data in the FHH section of EHR, and its performance was evaluated in comparison with gold standard annotation results. Next, we developed an NLP-augmented algorithm on top of the structured data algorithm (using only structured data) [8] to match the NCCN criteria using the NLP-processed results from both structured and unstructured fields. In the second stage, the performance of the NLP-augmented algorithm was compared with that of the structured data algorithm.

### Data Sets

**Figure 2:**
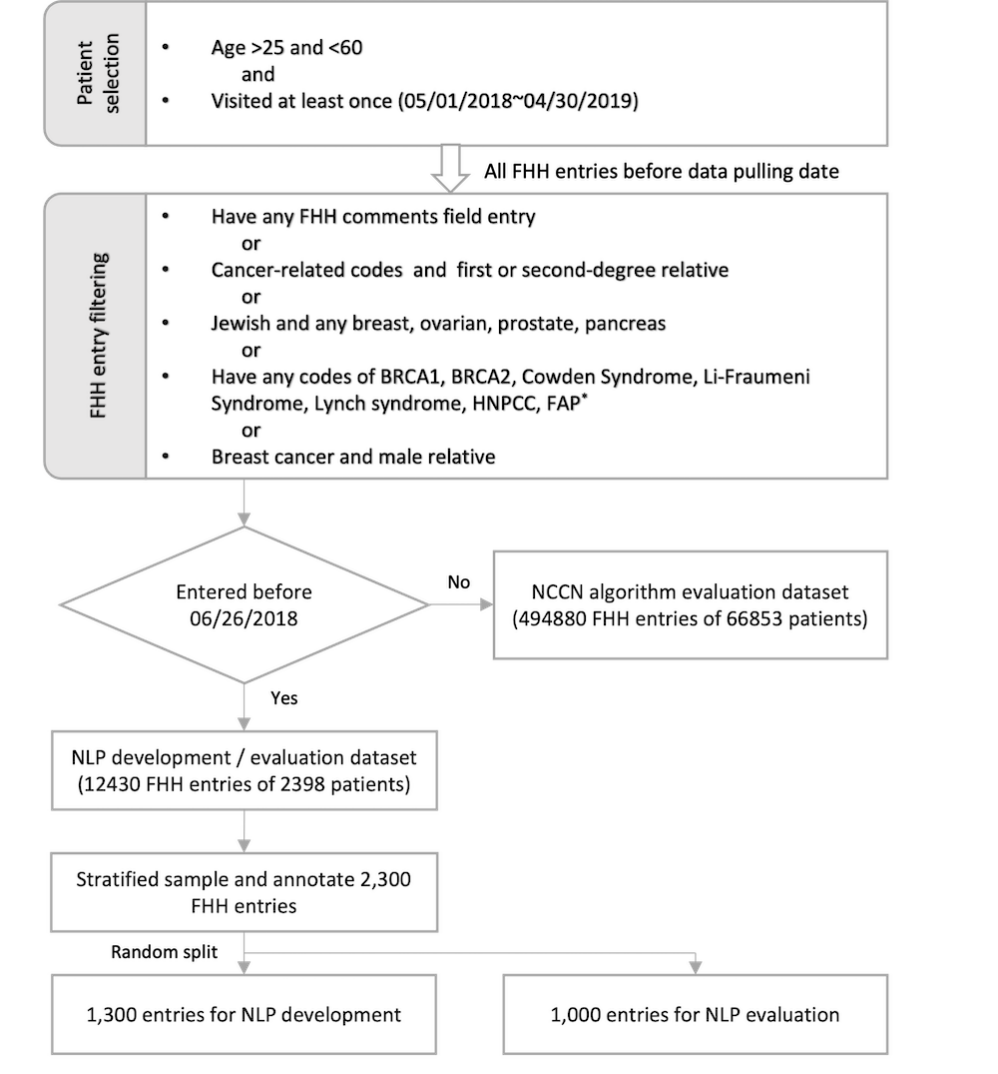
Data set creation process. FHH: family health history. NCCN: National Comprehensive Cancer Network. NLP: natural language processing. *HNPCC: hereditary non-polyposis colorectal cancer. FAP: familial adenomatous polyposis. Other genetic mutations or cancer syndromes specified in the NCCN guideline but without a code in electronic health record (EHR) were not included.

The data set for NLP development and evaluation consisted of EHR-based data from the FHH section (including both structured and unstructured fields) for 77,423 patients aged between 25 and 60 years who visited the University of Utah Health primary care clinic at least once between May 1, 2018, and April 30, 2019. All FHH entries of these patients were obtained, including entries recorded in prior visits to June 26, 2014. FHH entries contained a coded condition (breast cancer), a coded relative (sister), age of onset integer, and a free-text comment clinicians used to add detail (*in her 30s*). Entries that were not used to determine familial cancer risk were filtered using Structured Query Language (SQL), resulting in 31,191 entries. The detailed filtering strategy is illustrated in Figure 2.

The data set was split into 2. The FHH entries that were entered before June 26, 2018 were used for NLP development and evaluation (ie, the NLP development or evaluation data set), while entries entered after that date were used for algorithm evaluation (ie, the NCCN algorithm evaluation data set). We obtained a stratified random sample of 2300 FHH entries from the NLP development and evaluation data set. The stratification was based on the diagnosis codes in the condition field and stratified into four groups: (1) breast or ovarian cancer, (2) colorectal cancer, (3) other cancers, and (4) other noncancer family histories, at a 1:1:2:2 ratio. We randomly split 1300 FHH entries for NLP development, and the remaining 1000 entries were used for the snippet-level NLP evaluation. The NCCN algorithm evaluation data set was used to compare the performance of the 2 algorithms. Then, all the FHH entries (both data sets) were used to estimate the amount of additional information extracted by NLP and compare the patients identified by the NLP-augmented algorithm with those identified by the structured data algorithm.

### NLP Approach

#### Overview

Although NLP is often only used to process free-text data, independent of structured data, the comments field in the FHH section of EHR is used to supplement the structured data and cannot be interpreted in isolation. For example, in Table 1, the word *breast* supplements the concept *CANCER* in the structured condition field. Therefore, we concatenated the structure and comments fields into a single string for NLP processing. We also used double curly brackets to mark the values from the structured fields to reconcile conflicting information between the structured and comments fields (Table 1).

**Table 1:** An example of combining structured and unstructured data from FHH^a^ assertions.

Field names	Condition	Comments^b^	Family member	Age of onset
Original data	CANCER	Breast, great-aunt, dx at age of 52	AUNT	NULL
Combined	{{CANCER}}	Breast, great-aunt, dx at age of 52	{{AUNT}}	{{}}
Annotations	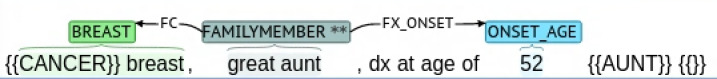			

^a^FHH: family health history.

^b^In this case, the comments field supplements or corrects the structured data, that is, CANCER is of the *breast*, and the family member (AUNT) is actually the patient’s great-aunt. FX_CANCER (FC): family member to cancer relationship; FX_ONSET: family member to age of onset relationship.

#### FHH Annotation Schema

**Figure 3:**
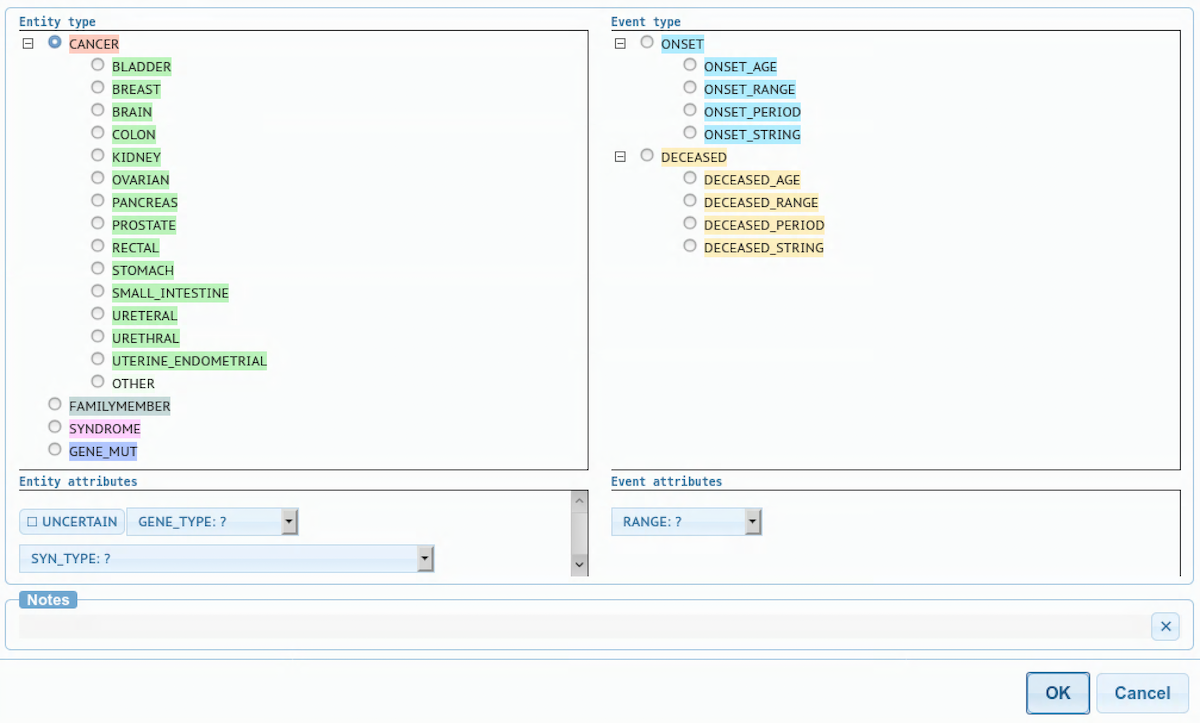
Screenshot of the schema as implemented with the annotation tool Brat.

A total of 2 physicians designed the annotation schema based on the FHH attributes relevant to the NCCN guidelines for genetic testing of hereditary breast or ovarian and colorectal cancers. This schema encompasses conditions, family members, and the age of onset. Specifically, the snippet-level data set contains (1) annotated entities for cancer diagnosis (CANCER), cancer-related syndromes (SYNDROME), cancer-related genetic mutations (GENE_MUT), family members (FAMILYMEMBER), and age of onset (ONSET), and (2) relations between family members and conditions, as well as between family members and age of onset. The example provided in Table 1 has 3 entities, that is, *great-aunt* (FAMILYMEMBER), ([CANCER]) *breast* (BREAST−breast cancer), *52* (ONSET_AGE), and 2 relations, that is, *great aunt* → *{{CANCER}} breast* (FX_CANCER) and *great aunt* → *52* (FX_ONSET). As the NCCN criteria include other cancers with mutations that share a common genetic pathway with breast, ovarian, and colorectal cancers, we added the following annotation subtypes: BLADDER, BREAST, BRAIN, COLON, KIDNEY, OVARIAN, PANCREAS, PROSTATE, RECTAL, STOMACH, SMALL_INTESTINE, URETERAL, and URETHRAL. As the NCCN criteria also use the side of the family of the affected family member and the degree of relationship, 2 attributes were included: family member CODE (eg, *GRANDMOTHER*) and SIDE of FAMILYMEMBER (eg, *PATERNAL*). In addition, an UNCERTAINTY feature was added to capture uncertainty statements (eg, *probably ovarian cancer).* We used a schema developed in our previous studies to annotate the age of onset [10], which includes 4 subtypes: ONSET_AGE (eg, *age 52*), ONSET_RANGE (eg, *in his 30s*), ONSET_PERIOD (eg, *in 1965*), and ONSET_STRING (eg, *postmenstruation*). Figure 3 presents a screenshot of the full schema within the annotation tool (Brat) [16]. The schema configuration is shared in GitHub [17].

#### NLP Development

**Figure 4:**
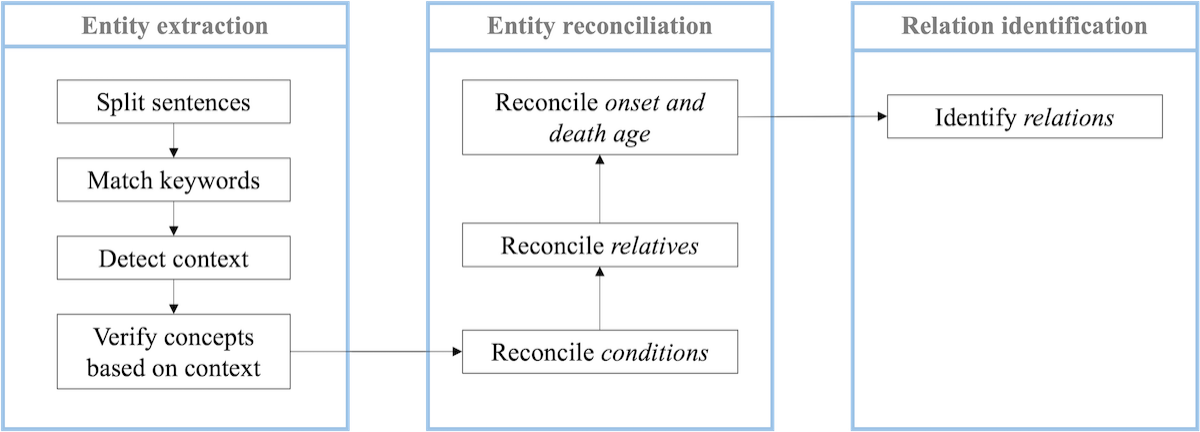
Easy clinical information extractor processing workflow. Three major steps (blue boxes): (1) entity extraction—extract the entities from the family health history entries; (2) entity reconciliation—reconcile the conflicts between the extracted entities; (3) relation identification—link related entities. In each step, there are ≥1 natural language processing components to complete processing substeps.

To develop the NLP pipeline, we used Easy clinical information extractor (EasyCIE), a lightweight rule-based NLP tool that supports rapid clinical NLP implementations [18]. All NLP components of EasyCIE are configurable through rules without the need to develop new pipelines. A total of 1300 FHH entries were used to develop the rules. We adopted a logic similar to that described by Goryachev et al [19] but implemented the logic in a different way for efficiency and generalizability considerations [20]. The processing consists of three major steps: (1) entity extraction, (2) entity reconciliation, and (3) relation identification (Figure 4). Each step was performed using one or more NLP components. The following paragraph explains these components using the examples in Table 1. Each component is configured using a separate rule set that incorporates a keyword dictionary or inference logic. These rules were developed based on 3 sources: Unified Medical Language System, training data set, and clinical domain experts’ input. The rule set is available on GitHub [21].

*Entity extraction* (step 1) extracts the key entities (5 types) from the FHH entries. First, we split the sentences if there were more than one sentence. Second, we attempted to match the input string with controlled vocabulary (a keyword dictionary). An example is shown in Table 1, *{{CANCER}}* breast was recognized as *BREAST* (cancer), *52* as *ONSET_AGE*, and *great aunt* and *AUNT* as *FAMILYMEMBER*. Next, we detected the double curly brackets around *AUNT*. These 2 symbols indicate the mention of *AUNT* was located in the structured field. Thus, we assigned the feature *is_structural* to *AUNT*. Finally, we verified the features of each entity to determine whether they matched any inference rules. In the example, a *FAMILYMEMBER* with the *is_structural* feature was classified as *STR_FAMILYMEMBER* (a family member in the structured field). This differentiation among entities in different contexts allows entity reconciliation in the next step. This component also allowed us to exclude irrelevant mentions of entities (eg, the *FAMILYMEMBER daughter* in the context of *live with her daughter*). Further details regarding the implementation of EasyCIE's rule-processing engine are available elsewhere [20].

*Entity reconciliation* (step 2) reconciles the extracted entities from the first step when conflicts exist between structured and unstructured data. The corresponding heuristic rules were iteratively developed based on annotated data from the training set with refinements based on error analysis after applying the algorithm to the training set. In addition, we obtained insights through discussions with clinical experts, who currently use the dedicated FHH section to document FHH. Specifically, the following (Textbox 2) heuristic rules were applied (Table 2, additional examples are listed).

**Heuristic rules.**

**Rules**

1. If the structured field indicated *colon cancer*, but the information in the comments field clarified the condition of interest to be colorectal cancer syndromes (eg, Lynch syndrome), SYNDROME overrode COLON (cancer) in the structured field
2. If the age of onset was documented as structured numeric data (eg, 50) but the comments field documented an *ONSET_RANGE* (eg, late 50s), the ONSET_RANGE overrode the structured age of onset
3. If the age of onset was available in structured data, and the comments field included *ONSET_PERIOD* (eg, in 1985) or *ONSET_STRING* (eg, 10 years ago), *ONSET_PERIOD* and *ONSET_STRING* >were ignored. (4) If no age of onset was documented in the structured field and the comments field included a *DECEASED_AGE*, the algorithm set an *ONSET_RANGE before* the *DECEASED_AGE*.
4. If the comments field contained information on a specific family member, the algorithm ignored the structured family member field unless the comments field included a conjunction such as *also* >or *and*. In the example in Table 1, the *FAMILYMEMBER great aunt* was likely a correction of the STR_FAMILYMEMBER *AUNT* >because the picklist associated with STR_FAMILYMEMBER did not include an option for *great aunt*. Thus, in the reconciliation, STR_FAMILYMEMBER *AUNT* is ignored.
5. If the comments field contained nonspecific family member information (eg, father’s side), whereas the structured field contained a specific family member, the structured field code was used, and information from the comments was added as attributes if applicable.
6. If a mention of *FAMILYMEMBER* was specified as multiple individuals (eg, 2× sisters), multiple instances of *FAMILYMEMBER* were created (eg, 2× sisters would lead to 2 instances).

**Table 2:** Heuristic rules to reconcile entities.

Structured fields	Example	Comments field	Example	Reconciliation
*COLON*^a^ (cancer)	{{CANCER, COLON}}	Colorectal cancer–related *SYNDROME*	Lynch syndrome	Chose *SYNDROME*
*ONSET_AGE*	{{50}}	*ONSET_RANGE*	The late 50s	Chose *ONSET_RANGE*
*ONSET_AGE*	{{50}}	*ONSET_PERIOD*	In 1985	Chose *ONSET_AGE*
*ONSET_AGE*	{{50}}	*ONSET_STRING*	10 years ago	Chose *ONSET_AGE*
NULL	{{}}	*DECEASED_AGE*	Deceased at age 60 years	Inferred the *ONSET_RANGE*
*FAMILYMEMBER*	{{AUNT}}	A specific *FAMILYMEMBER*	Great-aunt	Chose *FAMILYMEMBER* in comments
*FAMILYMEMBER*	{{MOTHER}}	A specific *FAMILYMEMBER* with conjunction statement	And grandmother	Use *FAMILYMEMBER* in both fields
*FAMILYMEMBER*	{{AUNT}}	Nonspecific	Father side	Chose *FAMILYMEMBER*, and added comments value as a feature, if applicable
NULL	{{}}	Multiple *FAMILYMEMBER*	2× sisters	Created two *FAMILYMEMBER* annotations

^a^Words in italics denote concepts in the NLP output according to the FHH annotation schema.

*Relation identification* (step 3) links related entities. In the example of Table 1, *great aunt* and *{{CANCER}} breast* were linked to create an *FX_CANCER* relation. It also linked *great aunt* and *52* to create an *FX_ONSET* relation. As *STR_FAMILYMEMBER AUNT* was changed to *IGN_FAMILYMEMBER*, *AUNT* in the structured field were not linked to *{{CANCER}} breast* or *52*. When counting the number of relatives of interest, the number of FAMILYMEMBER-CANCER relations was obtained instead of relative entities. For example, *ovarian and stomach cancer* in *grandmother* should be counted as 2 cancers in the NCCN criteria. Although the NLP algorithm generated one FAMILYMEMBER entity (grandmother), two FAMILYMEMBER-CANCER relations were generated. The same process is followed to handle cases where a single cancer assertion refers to multiple relatives, eg, *breast cancer in mother and aunt*.

#### NLP Performance Evaluation

We evaluated the NLP solution by comparing its output with the test set annotations of the snippet-level data set (1000 FHH entries). To save time and effort, entities with no relation were not annotated (eg, an entry that only has a condition without mentioning any family member); therefore, we did not evaluate the NLP performance for named entity recognition. Precision, recall, and F1 scores were calculated for relation identification. A true positive relation was counted when NLP-extracted information matched the reference standard for both the relation type and corresponding feature values, as well as the two linked entities. We applied the bootstrap sampling method [22] to estimate the 95% CI for each performance measurement and conducted error analyses by categorizing and counting different types of errors. Considering the mentions of *SYNDROME* (cancer syndrome) and *GENETIC_MUTATION* (cancer-related genetic mutation) were very rare in the data set, the CI for the performance related to the extraction of relations with these 2 entity types, that is, *FX_SYNDROME* (family member to cancer syndrome relation) and *FX_GENE_MUT* (family member to genetic mutation relation), could not be obtained. Thus, we only calculated the CIs of the microaverages of these 3 measurements using bootstrap methods over the aggregated data that included all 4 relation types.

#### Structured Data Algorithm for Patient Eligibility Assessment

A rule-based algorithm was previously developed [8] based on NCCN guidelines for the genetic testing of hereditary breast or ovarian and colorectal cancers [6,7] using only structured FHH data. The algorithm was implemented using an open-source CDS platform (OpenCDS [23]) through a standards-based approach based on CDS Hooks for Services and the Fast Healthcare Interoperability Resources standard for FHH data representation. On the basis of the patient’s age and FHH, the algorithm determines whether the patient meets the NCCN criteria for genetic testing. The algorithm has been deployed for clinical use and integrated with the Epic EHR at the University of Utah Health and New York University. The details of the algorithm and its deployment in clinical practice are available elsewhere [8,24]. In this study, we used a structured data algorithm as the baseline.

#### NLP-Augmented Algorithm

The NLP-augmented algorithm was built on top of the structured data algorithm by converting the NLP output into a structured FHH format (condition, family member, and age of onset). As a result, the same structured data algorithm consumes NLP-augmented data. To handle the uncertainties, 2 different NLP configurations were provided, including and excluding uncertainty assertions for each of the breast, ovarian, and colorectal cancer cohorts. The configuration that included cases with uncertainty assertions was used to estimate the impact of NLP augmentations on algorithm-identified genetic testing candidates.

#### NLP-Augmented Algorithm Evaluation

The evaluation of the NLP-augmented algorithm consisted of two parts: (1) comparing the performance of the NLP-augmented algorithm with that versus the structured data algorithm using manually reviewed data as a reference standard, and (2) estimating NLP’s impact on the patient cohort size generated by the structured data algorithm over the whole data set using the inclusion configuration. A patient-level data set was created in this study. Owing to the large size of the cohort, it was not feasible to validate the expected output for all patient cases. Therefore, we sampled and annotated the algorithm outputs (against the NCCN algorithm evaluation data set) instead of annotating the input data. A review of a subset of 200 cases showed that when the baseline and NLP-augmented algorithms agreed regarding patient eligibility for genetic testing, the algorithm outputs were correct in 100% of the cases. Therefore, for cost-efficient considerations, we applied stratified sampling to down-sample the cases in which the 2 algorithms agreed to maintain a 1:2 ratio between cases with agreement and disagreement. We sampled 100 cases in total, 50 breast and ovarian cancer screening candidates and 50 colorectal cancer screening candidates. Subsequently, 2 annotators independently reviewed these cases to determine whether the 2 algorithms reached the correct conclusion. Any disagreement between the 2 annotators was adjudicated by a third annotator.

The structured data and NLP-augmented algorithms were compared in terms of precision, recall, and F1 scores. The 95% CIs were computed using the bootstrap method. As we did not obtain the ground truth of the patients’ FHH by contacting the patients themselves, the reference standards were made solely based on the entries in the FHH section. Next, we estimated the effectiveness of NLP augmentation by comparing (1) the number of FHH entries that were computable for the NCCN criteria and (2) the number of patients who met the criteria with and without NLP.

### Ethics Approval

This study was approved by the institutional review board at the University of Utah (IRB_00154076).

## Results

### Data Set Description

After splitting the data set, 2398 patients with 12,430 FHH entries were included in the NLP development or evaluation data set and 66,853 patients with 494,880 FHH entries were included in the NCCN algorithm evaluation data set. A total of 8172 patients did not have any FHH entries and were excluded from the data set. These 2 data sets were similar in sex, race, ethnicity, and age (Table 3).

**Table 3:** Patient characteristics in the NLP^a^ development or evaluation data set and the NCCN^b^ algorithm evaluation data set.

Characteristic		NLP development or evaluation data set (n=2398)	NCCN algorithm evaluation data set (n=66,853)
Gender (male), n (%)		998 (41.2)	24,524 (36.7)
**Race, n (%)**			
	White	1752 (73.2)	51,171 (76.5)
	Other	359 (15)	9510 (14.2)
	Asian	141 (5.9)	2973 (4.4)
	Black or African American	67 (2.8)	1450 (2.2)
	Not reported	56 (2.3)	1226 (1.8)
	American Indian or Alaska Native	17 (0.7)	523 (0.8)
	Hispanic ethnicity	327 (13.6)	9147 (13.7)
Age (years), mean (SD)		40.2 (9.6)	42.6 (9.9)

^a^NLP: natural language processing.

^b^NCCN: National Comprehensive Cancer Network.

### NLP Performance Evaluation Results

Using the snippet-level test data set, we evaluated the NLP’s performance at the snippet level; the average precision was 0.94 with 95% CI 0.91-0.97, the average recall was 0.94 with 95% CI 0.90-0.96, the average F1 score was 0.94 with 95% CI 0.91-0.96. The performance of the measurements for each relationship type is presented in Table 4.

**Table 4:** The performance on the snippet-level data set.

Relation types	TP^a^	FP^b^	FN^c^	Precision	Recall	F1 score
FX_CANCER^d^	489	32	31	0.94	0.94	0.94
FX_SYNDROME^e^	2	1	3	0.67	0.40	0.50
FX_GENE_MUT^f^	2	0	0	1.00	1.00	1.00
FX_ONSET^g^	203	10	14	0.95	0.94	0.94
Microaverage^h^	N/A^i^	N/A	N/A	0.94 (0.91-0.97)	0.94 (0.90-0.96)	0.94 (0.91-0.96)

^a^TP: true positive.

^b^FP: false positive.

^c^FN: false negative.

^d^FX_CANCER: family member to cancer relation.

^e^FX_SYNDROME: family member to cancer syndrome relation.

^f^FX_GENE_MUT: family member to cancer-related gene-mutation relation.

^g^FX_ONSET: Family member to age of onset relationship.

^h^These scores were computed using aggregated data, including all 4 relation types. The CIs were computed using the bootstrap method.

^i^N/A: not applicable.

### NLP Error Analysis

On the basis of the snippet-level error analysis of the NLP output from the test data set of 1000 FHH entries, we found 6 error types (Table 5). Approximately 50% of the errors were not directly caused by NLP mistakes. The *Annotation Error* was made by the annotators, which is common when a large volume of data needs to be reviewed. In addition, as we only partially overlapped the annotations and adjudicated the disagreement between the annotators for greater efficiency, the data that were not overlapped might also have contributed to annotation errors. *Data Input Typos* were another complication, especially some rare typos; for example, *bladdler*. *Out of Vocabulary* signified the words and phrases that were not seen in the training set and not added to the knowledge base from Unified Medical Language System and experts’ suggestions. For instance, *precancer* in the entry of *{{CANCER, BREAST}} precancer, age 30 {{MOTHER}} {{}}* should override the breast cancer code, because *precancer* is a term that describes a lesion that may develop into cancer. The NLP did not recognize the term; therefore, it was not possible to exclude breast cancer as an existing family health history. A *Context Error* might happen when the context of the entities included subtleties that the NLP could not correctly parse, for example, *{{CANCER, COLON}} possible, colon cancer, died when pt was 5 years old {{FATHER}} {{}}*. The NLP did not expect that the *5-year old* was not describing the father’s age of onset in the comments field, but the patient’s age. Sometimes, the input data is so ambiguous (ambiguous input) that even our annotators were not sure of the exact meaning without referring to other sources. For example, the entry *{{CANCER, COLON}} ileum {{FATHER}} {{}}*, likely meant the father had *ileum cancer*, which overwrote *colon cancer*. However, we were not 100% confident if the father actually had both because most of the cases like these would have been coded as *{{CANCER, OTHER}} ileum {{FATHER}} {{}}*. In real practice, genetic counselors would need to go over some clinical notes to find statements that can be cross-referenced or reach out to the patient to confirm the information. These types of improper coding in the structured fields and the conflicting information between the structured fields and comments field indicate that the EHR user interface for FHH entry may benefit from redesign, such as allowing users to label uncertainty. Finally, when designing the schema for annotation, we aimed to capture as much useful information as possible. We included three aggregated types of cancer, *GYNECOLOGIC*, *GASTROINTESTINAL*, *GENITOURINARY*, to code cancers not specific to the anatomical sites indicated in the guidelines. However, when executing the algorithms, these types are less useful, as they would result in more false-positive cases that are likely not relevant to the requirements. Therefore, these 3 types were excluded from the final NLP solution. Compared with the snippet level, this *schema mismatch* caused errors. For instance, *colon rectal cancer* was annotated as *GASTROINTESTINAL* to capture both, but in the NLP implementation, only one RECTAL cancer was counted instead of two cancers to simplify the implementation. This mismatch did not affect the patient-level results but was counted as a snippet-level error.

**Table 5:** Type of snippet-level errors and counts.

Type of errors	False positive, n	False negative, n	Examples
Annotation error	10	13	A missed annotation
Data input typo^a^	1	5	bladdler ca^b^
Out of vocabulary^a^	2	6	Precancer
Context error^a^	22	11	Possible, colon cancer, died when pt was *5 years old* {{FATHER}}
Ambiguous input	2	3	{{CANCER, COLON}} ileum {{FATHER}}
Schema mismatch^c^	6	10	See above
Total	43	48	N/A^d^

^a^These 3 types of errors are natural language processing (NLP)–caused errors or can be fixed by improving the NLP.

^b^ca: cancer.

^c^This type of error does not need to be fixed.

^d^N/A: not applicable.

### NLP-Augmented Algorithm Evaluation Results

The first part of this evaluation compared the NLP-augmented algorithm (using the inclusion configuration) with the structured data algorithm over a stratified sample of 100 patients (50 breast cancer and 50 colorectal cancer, with a 1:2 ratio of cases with agreement versus disagreement between unstructured and structured data). The NLP-augmented algorithm performed better than the structured data algorithm both in precision (0.99, 95% CI 0.96-1.00 vs 0.81, 95% CI 0.65-0.95), recall (0.95, 95% CI 0.90-0.99 vs 0.29, 95% CI 0.19-0.40), and F1 scores (0.97, 95% CI 0.94-0.99 vs 0.43, 95% CI 0.31-0.54).

In the second part of this evaluation, using the whole data set, compared with the original structured FHH entries, NLP augmentation yielded 21,703 (33.6%) additional computable FHH entries, with 8692 (27.9%) entries added owing to the extraction of conditions, 2689 (69.3%) owing to age of onset, and 10,322 (34.9%) owing to family members. With these additional entries extracted by NLP, 1578 (51%) patients met the NCCN criteria for breast cancer genetic testing, 373 (94%) patients met the criteria for colorectal cancer genetic testing, and 1841 (53.8%) additional unique patients met either or both criteria.

## Discussion

### Principal Findings

This study developed and evaluated an NLP-augmented algorithm to identify patients who met evidence-based criteria for genetic testing of hereditary colorectal and breast cancer. Overall, the proposed automated algorithm offers a promising approach to identifying these patients as an alternative to current clinical workflows, which rely on extensive manual review of patient records. We also demonstrated that compared with structured data alone, an NLP algorithm that focused on the interplay between structured data and associated free-text comments significantly increased the computability of FHH entries and algorithm accuracy. Compared with structured data alone, NLP augmentation led to a 53.8% increase in the number of patients available to compute against the NCCN criteria for genetic testing.

Chen et al recognized the significance of data recorded in the FHH section of an EHR [12]. They characterized the use and contents of the FHH comments field and found that it was used to augment or modify the attributes of the statement (eg, uncertainty and negation) for all 3 types of entities: *family member*, *condition*, and *age of onset*. However, they did not develop a complete solution for extracting these relationships. In a previous study, we used NLP to extract the disease age of onset from the comments field [10]. In this study, we extended the NLP solution to extract all 3 types of entities and the relations between them. In addition, the algorithm reconciles information from structured and unstructured data to identify patients who meet the NCCN criteria for genetic testing of 2 common hereditary cancers. The study results demonstrated that the NLP-augmented algorithm accurately extracted relevant FHH at the snippet level that combined the structured and comments fields. At the patient level, the algorithm significantly improved the recall and precision of identifying patients who met the NCCN criteria for genetic testing of hereditary breast colorectal cancer.

Compared with previously published studies on FHH extraction using NLP, this study differs significantly in the input data source, types of technical challenges, and ultimate goals. Previous studies have focused primarily on extracting FHH from clinical notes, whereas our approach targets the FHH section of the EHR by combining structured and unstructured data. Complete sentences are typical in the FHH narrative of clinical notes, while single words, phrases, and short sentences are more typical in the FHH comment fields. Consequently, the technical challenges are different. Challenges in extracting FHH from clinical notes include FHH section detection, entity recognition, and relation detection [9,14,15]. In contrast, targeted extraction from the FHH section of the EHR requires reconciliation between structured and unstructured data, as they can be complementary, redundant, or conflicting [12]. In addition, extraction from clinical notes focuses on general FHH extraction, whereas our approach aims to identify patients with a specific clinical purpose. Thus, the NLP performance reported in Table 4 is not directly comparable with that reported in previous studies.

As noted above, the NLP-augmented algorithm can be configured to include or exclude FHH entries with uncertain statements in the free-text comments. The choice of configuration depends on the requirements of specific use cases and available institutional resources. For instance, in a study that aimed to reach out to eligible patients offering genetic testing, a higher priority may have been given to patients who met testing criteria with a higher degree of certainty (ie, excluding uncertain statements) to minimize manual screening efforts. In contrast, if genetic testing outreach is rolled out as usual care, an institution may want to maximize the benefits of genetic testing to as many patients as possible by including uncertain statements. The difference in algorithm performance between the 2 configurations (ie, including vs excluding uncertainty statements) was not significant. Thus, we did not report the results using the exclusion configuration.

The results showed that the NLP-augmented algorithm had significantly higher precision and recall than structured data alone in identifying patients who met the NCCN criteria for genetic testing. This increase was achieved because the comments field provided additional information that can be used to compute the NCCN criteria, including the cancer type (eg, *pancreatic cancer)*, the age of onset (eg, *diagnosed colon cancer, at age 40*), and the affected family member (eg, *paternal aunt*). In addition, information in the comments field can correct inaccurate data in structured fields.

### Limitations

This study had several limitations. First, we used data from one EHR at an academic medical center. Therefore, we cannot conclude that the algorithm and study findings are generalizable to other EHRs and health care systems. However, the EHR used in this study is one of the most widely used EHRs in the United States, and other EHR products use similar FHH sections to collect FHH data [12], suggesting that the proposed approach may be adapted to those settings. Second, error analysis demonstrated that certain FHH entries could not be disambiguated based on the available data provided in the FHH section. Future studies could investigate approaches to disambiguate these FHH entries, such as applying NLP to clinical notes or asking patients to confirm through the patient portal.

As the patient-level data set down-sampled the cases in which the 2 algorithms agreed, the difference between the NLP-augmented algorithm and the structured data algorithm was amplified correspondingly. Thus, we did not analyze the statistical differences between the algorithms on this data set. Despite this, the results showed that when these 2 algorithms disagreed with each other, the NLP-augmented algorithm likely received correct answers. In addition, because of the down-sampling, more challenging cases were likely included in the reference data set compared with the original data set. Thus, the actual performance of both algorithms is potentially higher than the scores reported in the section of *NLP-Augmented Algorithm Evaluation Results*.

Although the NLP-augmented algorithm still missed eligible patients, it achieved higher recall than the structured algorithm. Future studies could investigate combining FHH extraction from both FHH sections and clinical notes to further reduce false-negative errors. In addition, other solutions beyond NLP are needed to improve the accuracy and comprehensiveness of the FHH collection in the EHR.

Finally, we investigated only a rule-based solution for the NLP task. Given that the performance was satisfactory and the rule-based approach could be customized quickly for error fixing and future enhancements, we decided that it was not worthwhile to investigate more complex machine learning–based solutions.

### Conclusions

This study demonstrated that our NLP solution can accurately extract FHH from both the structured and unstructured fields of the FHH section. Applying this NLP solution to augment the structured data algorithm could improve the precision and recall of identifying patients who meet the NCCN criteria for genetic testing of hereditary breast and colorectal cancer.

## Abbreviations

CDS: clinical decision support
EasyCIE: easy clinical information extractor
EHR: electronic health record
FHH: family health history
NCCN: National Comprehensive Cancer Network
NLP: natural language processing
SQL: Structured Query Language
