# Correction: Identifying Patients Who Meet Criteria for Genetic Testing of Hereditary Cancers Based on Structured and Unstructured Family Health History Data in the Electronic Health Record: Natural Language Processing Approach

**DOI:** 10.2196/42533

**Published:** 2022-09-13

**Authors:** Jianlin Shi, Keaton L Morgan, Richard L Bradshaw, Se-Hee Jung, Wendy Kohlmann, Kimberly A Kaphingst, Kensaku Kawamoto, Guilherme Del Fiol

**Affiliations:** 1 Veterans Affairs Informatics and Computing Infrastructure Department of Veterans Affairs Salt Lake City Health Care System Salt Lake City, UT United States; 2 Division of Epidemiology, Department of Internal Medicine School of Medicine University of Utah Salt Lake City, UT United States; 3 Department of Biomedical Informatics University of Utah Salt Lake City, UT United States; 4 Department of Emergency Medicine University of Utah Salt Lake City, UT United States; 5 College of Nursing University of Utah Salt Lake City, UT United States; 6 Department of Population Health Sciences University of Utah Salt Lake City, UT United States; 7 Huntsman Cancer Institute University of Utah Salt Lake City, UT United States; 8 Department of Communication University of Utah Salt Lake City, UT United States

In “Identifying Patients Who Meet Criteria for Genetic Testing of Hereditary Cancers Based on Structured and Unstructured Family Health History Data in the Electronic Health Record: Natural Language Processing Approach” (JMIR Med Inform 2022;10(8):e37842), the authors noted the following corrections:

(1) In the originally published paper, the following sentence was present in the *Methods* section of the *Abstract*:

Algorithms were developed based on the National Comprehensive Cancer Network (NCCN) guidelines for genetic testing for hereditary breast or ovarian and colorectal cancers.

This has been changed to:

Algorithms were developed based on the National Comprehensive Cancer Network (NCCN) guidelines for genetic testing for hereditary breast, ovarian, pancreatic, and colorectal cancers.

(2) Textbox 1 has been revised for clarity and accuracy, and to comply with the citation guidelines of the National Comprehensive Cancer Network (NCCN).

(3) In the originally published paper, the following sentence was present in the *Background* section:

The National Comprehensive Cancer Network (NCCN) has published a set of evidence-based guidelines for genetic testing of hereditary cancers, including breast, ovarian, and colorectal cancers.

This has been changed to:

The National Comprehensive Cancer Network (NCCN) has published a set of evidence-based guidelines for genetic testing of hereditary cancers, including breast, ovarian, pancreatic, and colorectal cancers.

(4) References [6] and [7] have been updated to the following with NCCN's permission and disclaimer statements:

6. Daly MB, Pilarski R, Yurgelun MB, Berry MP, Buys SS, Dickson P, et al. NCCN guidelines insights: genetic/familial high-risk assessment: breast, ovarian, and pancreatic, version 1.2020. J Natl Compr Canc Netw 2020 Apr;18(4):380-391 [Referenced with permission from the National Comprehensive Cancer Network, Inc. 2020]. [doi: 10.6004/jnccn.2020.0017] [Medline: 32259785]

7. Gupta S, Provenzale D, Llor X, Halverson AL, Grady W, Chung DC, et al. NCCN guidelines insights: genetic/familial high-risk assessment: colorectal, version 2.2019. J Natl Compr Canc Netw 2019 Sep 01;17(9):1032-1041 [Referenced with permission from the National Comprehensive Cancer Network, Inc. 2020]. [doi: 10.6004/jnccn.2019.0044] [Medline: 31487681]

**Excerpt of National Comprehensive Cancer Network (NCCN) criteria for unaffected individuals’ family history–based genetic testing of breast, ovarian, pancreatic, and colorectal cancers (referenced with permission).**

**Breast or ovarian cancer:**

1. First- or second-degree relative with breast cancer at age ≤45 years
2. First- or second-degree relative with ovarian cancer
3. First-degree relative with pancreatic cancer
4. Breast cancer in a male relative
5. Three or more first- or second-degree relatives with breast or prostate cancer on the same side of the family
6. Ashkenazi Jewish and any breast or prostate cancer in any relative at any age
7. BRCA1/2, CHEK2, ATM, PALB2, TP53, PTEN, or CDH1 genes, Cowden Syndrome, Li-Fraumeni Syndrome in any relative at any age

**Colorectal cancer:**

1. MLH1, MSH2, PMS2, MSH6, EPCAM, MYH, or MUTYH genes, Lynch syndrome, familial adenomatous polyposis (FAP), adenomatous polyposis coli (APC), serrated polyposis or polyposis discovered in the coded family history
2. First-degree relative with colon cancer at ≤50 years
3. First-degree relative with endometrial cancer at ≤50 years
4. Three or more first- or second-degree relatives with Lynch syndrome, HNPCC, colon cancer, endometrial, uterine, ovarian, stomach, gastric, small bowel, small intestine, kidney, ureteral, bladder, urethra, brain, pancreas, also all on the same side of the family

The correction will appear in the online version of the paper on the JMIR Publications website on September 13, 2022, together with the publication of this correction notice. Because this was made after submission to PubMed, PubMed Central, and other full-text repositories, the corrected article has also been resubmitted to those repositories.
